# Plasma Metabolite Profiles of Red Meat, Poultry, and Fish Consumption, and Their Associations with Colorectal Cancer Risk

**DOI:** 10.3390/nu14050978

**Published:** 2022-02-25

**Authors:** Fenglei Wang, Paulette D. Chandler, Oana A. Zeleznik, Kana Wu, You Wu, Kanhua Yin, Rui Song, Julian Avila-Pacheco, Clary B. Clish, Jeffrey A. Meyerhardt, Xuehong Zhang, Mingyang Song, Shuji Ogino, I-Min Lee, A. Heather Eliassen, Liming Liang, Stephanie A. Smith-Warner, Walter C. Willett, Edward L. Giovannucci

**Affiliations:** 1 Department of Nutrition, Harvard T.H. Chan School of Public Health, Boston, MA 02115, USA; fengleiwang@g.harvard.edu (F.W.); hpkwu@channing.harvard.edu (K.W.); yow728@g.harvard.edu (Y.W.); ruisong@g.harvard.edu (R.S.); poxue@channing.harvard.edu (X.Z.); mis911@mail.harvard.edu (M.S.); swarner@hsph.harvard.edu (S.A.S.-W.); wwillett@hsph.harvard.edu (W.C.W.); 2 Division of Preventive Medicine, Department of Medicine, Brigham and Women’s Hospital and Harvard Medical School, Boston, MA 02115, USA; pchandler@bwh.harvard.edu (P.D.C.); ilee@rics.bwh.harvard.edu (I.-M.L.); 3 Channing Division of Network Medicine, Brigham and Women’s Hospital and Harvard Medical School, Boston, MA 02115, USA; nhotz@channing.harvard.edu (O.A.Z.); nhahe@channing.harvard.edu (A.H.E.); 4 Department of Epidemiology, Harvard T.H. Chan School of Public Health, Boston, MA 02115, USA; yinkanhua@gmail.com (K.Y.); sogino@bwh.harvard.edu (S.O.); lliang@hsph.harvard.edu (L.L.); 5 Broad Institute of MIT and Harvard, Cambridge, MA 02142, USA; jravilap@broadinstitute.org (J.A.-P.); clary@broadinstitute.org (C.B.C.); 6 Dana-Farber Cancer Institute and Harvard Medical School, Boston, MA 02215, USA; jeffrey_meyerhardt@dfci.harvard.edu; 7 Clinical and Translational Epidemiology Unit, Massachusetts General Hospital and Harvard Medical School, Boston, MA 02114, USA; 8 Division of Gastroenterology, Massachusetts General Hospital, Boston, MA 02114, USA; 9 Program in MPE Molecular Pathological Epidemiology, Department of Pathology, Brigham and Women’s Hospital and Harvard Medical School, Boston, MA 02115, USA; 10 Cancer Immunology Program, Dana-Farber Harvard Cancer Center, Boston, MA 02215, USA; 11 Department of Biostatistics, Harvard T.H. Chan School of Public Health, Boston, MA 02115, USA

**Keywords:** red meat, fish, plasma metabolomics, colorectal cancer

## Abstract

Background: Red and processed meat consumption has been consistently associated with increased risk of colorectal cancer (CRC), but the association for fish intake is unclear. Evidence using objective dietary assessment approaches to evaluate these associations is sparse. Objectives: We aim to investigate the plasma metabolite profiles related to red meat, poultry, and fish consumption and examine their associations with CRC risk. Methods: We measured plasma metabolites among 5269 participants from the Nurses’ Health Study (NHS), NHSII, and Health Professionals Follow-Up study (HPFS). We calculated partial Spearman correlations between each metabolite and self-reported intake of seven red meat, poultry, and fish groups. Metabolite profile scores correlated to self-reported dietary intakes were developed using elastic net regression. Associations between self-reported intakes, metabolite profile scores, and subsequent CRC risk were further evaluated using conditional logistic regression among 559 matched (1:1) case-control pairs in NHS/HPFS and replicated among 266 pairs in Women’s Health Study. Results: Plasma metabolites, especially highly unsaturated lipids, were differentially associated with red meat and fish groups. Metabolite profile scores for each food group were significantly correlated with the corresponding self-reported dietary intake. A higher dietary intake of processed red meat was associated with a higher risk of CRC (pooled OR per 1 SD, 1.15; 95% CI: 1.03, 1.29). In contrast, higher metabolite profile scores for all fish groups, not dietary intakes, were consistently associated with a lower CRC risk: the pooled OR per 1 SD was 0.86 (95% CI: 0.78, 0.96) for total fish, 0.86 (95% CI: 0.77, 0.96) for dark meat fish, and 0.87 (95% CI: 0.78, 0.97) for canned tuna fish. No significant associations were found for other food groups. Conclusions: Red meat and fish intake exhibited systematically different plasma metabolite profiles. Plasma metabolite profile of fish intake was inversely associated with CRC risk.

## 1. Introduction

Colorectal cancer (CRC) remains the second most commonly occurring cancer in women and the third in men worldwide [1]. CRC incidence is largely affected by screening and modifiable dietary and lifestyle factors [2]. Among these factors, meat and fish consumption has been the subject of many investigations. The consumption of red meat, especially processed red meat, is consistently associated with an increased risk of CRC [3]. However, there is still uncertainty regarding the association between fish intake and CRC risk. The World Cancer Research Fund and American Institute for Cancer Research (WCRF/AICR) concluded that there was only “limited evidence” for the beneficial effect of fish intake in CRC prevention [4]. Recent studies of fish intake and CRC risk also generated inconsistent results [5,6,7,8].

Previous epidemiological studies examining the association of meat and fish intake with CRC risk mainly used self-reported dietary data. Rapid developments in high-throughput metabolomics are leading to a new era in nutritional epidemiological research. By measuring the small-molecule metabolites in biological samples, metabolomics may provide an objective picture of food intake and its related biological consequences [9]. Feeding trials and observational studies have demonstrated that plasma and urinary metabolites differed between meat and fish consumption [10,11,12,13,14]. However, it is unknown whether the metabolite profiles related to meat and fish intake are associated with CRC risk and whether these profiles could be used as a complementary approach to evaluate the association between meat and fish intake and CRC risk.

Therefore, we examined the associations of red meat, poultry, and fish consumption with plasma metabolite profiles among participants from the Nurses’ Health Study (NHS), NHSII, and Health Professionals Follow-up Study (HPFS). We also developed metabolite profile scores that were correlated to the intakes of these meat and fish groups and evaluated the prospective associations of metabolite profile scores with CRC risk. The results for metabolite profile scores were further replicated in an external validation cohort (Women’s Health Study, WHS).

## 2. Methods

### Study Population

Our primary analyses were based on three prospective cohorts: NHS, NHSII, and HPFS. The NHS started in 1976 among 121,700 female registered nurses aged 30–55 years, and the NHSII began in 1989 among 116,429 younger female registered nurses aged 25–42 years [15]. The HPFS was initiated in 1986 and enrolled 51,529 male health professionals aged 40–75 years [16]. Blood samples were collected from subsamples of the NHS between 1989 and 1990, NHSII between 1996 and 1999, and HPFS between 1993 and 1995 [17,18]. We included participants who provided blood samples and were previously selected for metabolomics sub-studies of breast cancer and CRC (all diagnosed after blood collection). After excluding participants with missing dietary data, 5269 participants (2627 from NHS, 2096 from NHSII, and 546 from HPFS) were included in the final analyses. Among them, we included 559 case-control pairs (404 pairs for colon cancer and 122 pairs for rectal cancer) in the analysis of CRC risk. Each CRC case was matched to one control by age, month, and fasting status at blood collection.

The external replication analysis was performed in WHS, a completed randomized controlled trial originally designed to examine the role of aspirin and Vitamin E in the prevention of cancer and cardiovascular disease [19]. From 1992 to 1995, 39,876 healthy women aged 45 years or older were recruited. Blood samples were provided by 71% of participants before randomization. The trial ended in 2004, but annual observational follow-up continued. We included samples from 266 CRC cases (diagnosed after blood collection) and matched controls with available dietary and metabolomics data. Each case was matched to a control by age, ethnicity, month, and fasting status at the time of blood collection and added follow-up time [20]. The study protocol was approved by the institutional review boards of the Brigham and Women’s Hospital and Harvard T.H. Chan School of Public Health and those of participating registries as required.

## 3. Dietary Assessment

Validated semi-quantitative food frequency questionnaires (FFQs) were administered to assess long-term intake of foods and nutrients in NHS/NHSII/HPFS every four years [21,22,23]. Participants were asked to report how often, on average, they consumed a standard portion size of each food in the past year. To better reflect dietary consumption at the time of blood draw, we calculated the average intakes from the two FFQs closest to the blood collection date for each cohort (1986 and 1990 in NHS, 1995 and 1999 in NHSII, and 1990 and 1994 in HPFS). We included seven meat and fish groups in the analyses: total red meat, unprocessed red meat, processed red meat, poultry, total fish, dark meat fish, and canned tuna fish. Consistent with previous studies from the same cohorts [24], unprocessed red meat included hamburgers; beef, pork, or lamb as a sandwich or mixed dish; and beef, pork, or lamb as a main dish. Processed red meat included bacon; hot dogs; and sausage, salami, bologna, or other processed meat. Total red meat was derived by summing consumption of unprocessed and processed red meat. Poultry included chicken or turkey with or without skin; chicken or turkey sandwiches; and chicken or turkey hot dogs. Total fish included dark meat fish (e.g., salmon); canned tuna fish; breaded fish cakes, pieces, or fish sticks; and other fish. In WHS, dietary information was collected using the same validated FFQ at trial baseline. The serving sizes for each food group are shown in Supplementary Table S1.

## 4. Metabolomics Measurement

Profiles of plasma metabolites in all four studies (NHS, NHSII, HPFS, and WHS) were obtained using high-throughput liquid chromatography-mass spectrometry techniques at the Broad Institute of MIT and Harvard [25]. Hydrophilic interaction liquid chromatography with positive ion mode mass spectrometry detection was used to separate polar metabolites, and C8 chromatography with positive ion mode detection was used to profile lipids. Raw data were processed using TraceFinder software (Thermo Fisher Scientific Waltham, MA, USA) and Progenesis QI (Nonlinear Dynamics, Newcastle upon Tyne, UK). Known metabolite identities were confirmed using authentic reference standards or reference samples. Unknown metabolites were aligned using an in-house alignment algorithm, m2Aligner. This tool identifies unambiguous shared peaks in datasets to be aligned and uses them as alignment vectors adjusting for deviations in retention time (RT), mass to charge ratio (m/z), and abundance for all the peaks in the datasets. The adjusted m/z and RTs are subsequently used to match peaks using a scoring system that takes into consideration their mass accuracy and their retention time deviation (the corresponding methodology manuscript is in preparation).

We excluded known or unknown metabolites whose intraclass correlation coefficient (ICC) across blinded quality control replicates (10% of study sample) were <0.4, had no between-person variation, or detection rate <75%. Metabolites that were not stable with the processing delay inherent in our cohort study blood collections were also excluded (*n* = 38) [26]. Metabolite levels were reported as measured LC-MS peak areas, which are proportional to metabolite concentration. Metabolite peak areas were then log-transformed and converted to z-scores with a mean of 0 and a standard deviation of 1 within each sub-study. A total of 287 known and 2561 unknown metabolites were included in the present analyses. Among these metabolites, 58 known metabolites had missing data, with a missing proportion ranging from 0.02% to 13.5%; 1206 unknown metabolites had missing data, with a missing proportion ranging from 0.02% to 22.1%. The missingness was imputed using ½ minimum value. The 287 known metabolites were primarily lipids (*n* = 206, including 85 glycerolipids, 31 glycerophospholipids, 22 plasmalogens, 21 carnitines, 21 lysophospholipids, 13 cholesterol & cholesterol esters, 5 sphingolipids, 4 steroids, and 4 ceramides), but also included amino acids related metabolites (*n* = 41) and other metabolites (*n* = 40) (Supplementary Figure S1A). The lipid metabolites were highly correlated with each other within categories (Supplementary Figure S1B). A majority of the known metabolites (254 out of 287) were qualified for replication analysis in WHS. Metabolite data were log-transformed and converted to z-scores within each sub-study.

## 5. Nondietary Covariates

In NHS/NHSII/HPFS, we collected information on lifestyle factors, including smoking, physical activity, multivitamin use, aspirin use, history of previous endoscopy, and family history of CRC using the biennial follow-up questionnaires. BMI (in kg/m^2^) was calculated using height reported at baseline and body weight reported closest to the blood draw. Age and fasting status were collected via questionnaires completed at blood collection. In WHS, participants provided information on age, weight, height, and lifestyle factors at baseline.

### Statistical Analyses

We examined the associations between intake of the seven meat and fish groups and each known and unknown metabolite in NHS/NHSII/HPFS, using partial Spearman correlation analysis adjusting for age and fasting status at blood draw, endpoint, and case/control status in the original sub-study, smoking, BMI, physical activity, total energy intake, alcohol intake, and modified Alternate Healthy Eating Index (AHEI, a measure of diet quality; intakes of red meat, alcohol, trans fat, long-chain n-3 fats, and polyunsaturated fats were not included in the calculation). Intakes of red meat, poultry, and fish were mutually adjusted. The Benjamini-Hochberg false discovery rate (FDR) and Bonferroni correction were used to account for multiple testing.

**Figure 1:**
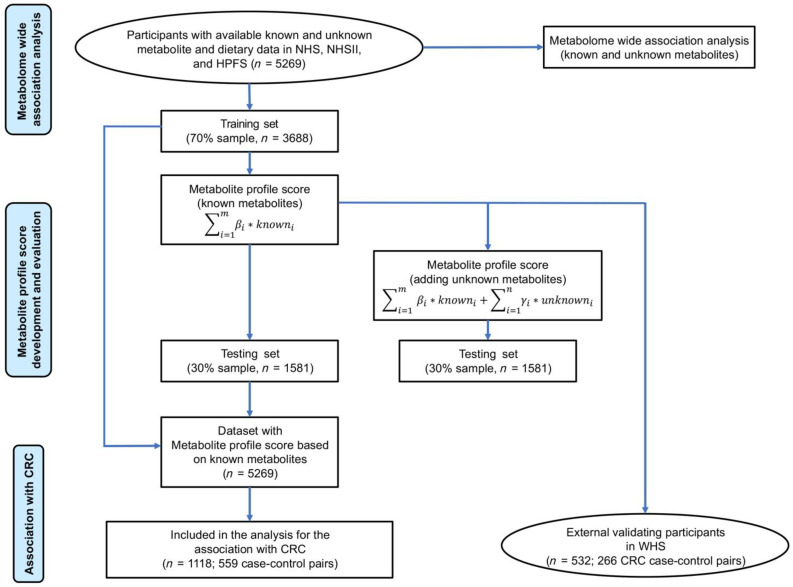
Schematic of the study design for the metabolome wide association analysis, development and evaluation of the metabolite profile scores, and analysis of CRC risk. CRC, colorectal cancer; HPFS, Health Professionals Follow-up Study; NHS, Nurses’ Health Study; NHSII, Nurses’ Health Study II; WHS, Women’s Health Study.

To develop metabolite profile scores that are correlated to the consumption of seven meat and fish groups, NHS/NHSII/HPFS participants were randomized to either the training set (*n* = 3688) or the testing set (*n* = 1581) in a 7 to 3 fashion (Figure 1). The dietary consumption data were inverse normal transformed to improve normality. We used an elastic net [27] with 10-fold cross-validation to regress the consumption of each meat and fish group on the 287 known metabolites in the training set. The trained model was then applied to calculate the metabolite profile score for the testing set and participants in WHS. The metabolomic score was calculated as the weighted sum of the selected metabolites with weights equal to coefficients from the elastic net regression. Metabolite profile scores in the training set were obtained using a leave-one-out approach to avoid overfitting. Pearson correlation coefficient between self-reported dietary consumption and the corresponding metabolite profile score was calculated to evaluate how well the score was correlated to the dietary consumption. Apart from the selected known metabolites in the metabolite profile scores, we added unknown metabolites into the elastic net model and developed a new score that included the unknown ones (Figure 1). The new metabolomic scores were applied to the testing set, and their Pearson correlation coefficients with the corresponding dietary consumption were calculated as well. We then compared the Pearson correlation coefficients before and after including unknown metabolites to assess the contribution of unknown metabolites to the correlation between metabolomic score and dietary consumption [28].

Associations of meat and fish consumption and their metabolite profile scores with CRC were assessed among 559 pairs of CRC cases and matched controls from NHS/HPFS and 266 pairs from WHS (Figure 1). Conditional logistic regression adjusting for BMI, family history of CRC, history of endoscopy, multivitamin use, aspirin use, smoking, physical activity, alcohol intake, total energy intake, and modified AHEI was used to estimate the odds ratios (OR) and 95% confidence intervals (CI) for one SD increase in dietary intake or metabolite profile score. These covariates for adjustment were selected based on our subjective knowledge and previous analysis results in the cohorts. Results from NHS/HPFS and WHS were then pooled using a fixed-effect model. All statistical analyses were performed in R version 4.1.

## 6. Results

In NHS/NHSII/HPFS, participants were predominately white and middle-aged (mean age 53 years), with an average BMI of 25.4 kg/m^2^ (Table 1). The percentage energy intake from protein was 18%, and animal protein was 13%. Participants with a higher total red meat intake were slightly more likely to have a lower total fish intake (Pearson *r* = −0.07). In the analysis of CRC, participants had similar demographic characteristics but were a little older (mean age 61 years) and had a lower proportion of females. Compared to control participants, CRC cases were less likely to use multivitamins and aspirin, receive endoscopy screening, and be physically active, but were more likely to smoke and have a family history of CRC. They also had a somewhat higher intake of total red meat (mainly processed red meat) and a lower fish intake. Similarly, CRC cases in WHS were less likely to be physically active and more likely to smoke and have a family history of CRC. However, they had a lower intake of total red meat (mainly unprocessed red meat) than control participants.

**Figure 2:**
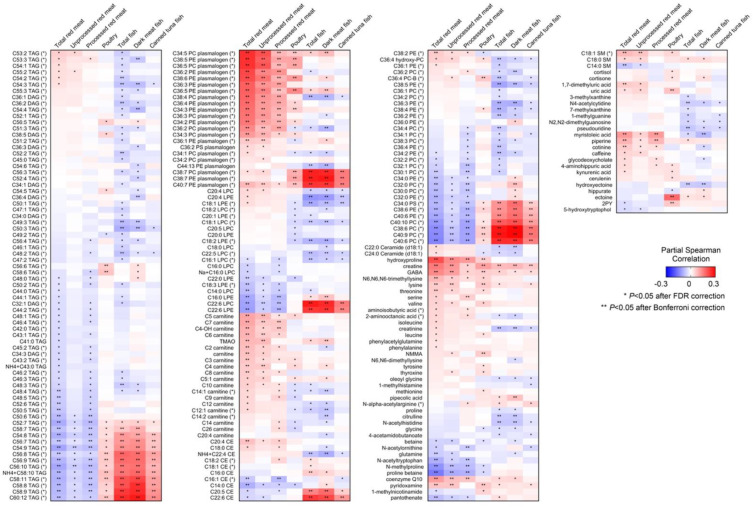
Heatmap of known metabolites that are significantly associated with meat and fish intake. Only metabolites significantly (after FDR correction) correlated with at least one meat or fish group are shown. The intensity of the colors represents the degree of association between plasma metabolites and consumption of total red meat, unprocessed red meat, processed red meat, poultry, total fish, dark meat fish, and canned tuna fish, as measured by partial Spearman correlation analyses adjusting for age at blood draw, fasting status, endpoints, and case/control status in the original sub-study, BMI, smoking, physical activity, alcohol intake, total energy intake, and modified AHEI. These meat and fish groups were also mutually adjusted. Metabolite with “(*)” indicate a representative name. *, *p* < 0.05 after FDR correction and **, *p* < 0.05 after Bonferroni correction. AHEI, Alternate Healthy Eating Index; BMI, body mass index.

Of the 287 known metabolites, 85 were significantly correlated with total red meat intake, 36 with unprocessed red meat, 51 with processed red meat, 28 with poultry, 60 with total fish, 60 with dark meat fish, and 27 with canned tuna fish after Bonferroni correction (Figure 2). Similar metabolites correlated with total red meat intake, unprocessed red meat, and processed red meat. All three red meat groups were positively associated with creatine, hydroxyproline, coenzyme Q10, myristoleic acid, acylcarnitines, and plasmalogens with a number of double bonds ≤6 but were negatively associated with highly unsaturated lipid species including triglycerides (TAGs), phosphatidylethanolamines (PEs), phosphatidylcholines (PCs), lysophosphatidylethanolamines (LPEs), and lysophosphatidylcholines (LPCs). Poultry intake was positively correlated with creatine, ectoine, a few PCs, TAGs, and PE plasmalogens after Bonferroni correction. In contrast to red meat intake, total fish intake, dark meat fish, and canned tuna fish were all positively correlated with highly unsaturated lipid species. When we restricted the analysis to fasting participants and participants selected as controls in the original sub-studies, the correlation results were similar (Supplementary Figures S2 and S3).

Of the 2561 unknown metabolites, 702 were significantly correlated with total red meat intake after Bonferroni correction, 222 with unprocessed red meat, 462 with processed red meat, 65 with poultry, 284 with total fish intake, 414 with dark meat fish, and 119 with canned tuna fish (Supplementary Table S2). Among these, the absolute partial Spearman *rho* exceeded 0.3 for 11 unknown metabolites: one with total red meat intake, 10 with total fish intake, and 5 with dark meat fish intake. Pearson correlation analysis between these 11 unknown metabolites and those known metabolites (130 in total) that were significantly associated with total red meat, total fish, or dark meat fish consumption identified several unknown metabolites being strongly correlated with C38:6 PC, C40:6 PC, C40:9 PC, and C22:6 LPC (all Pearson *r* > 0.8) (Supplementary Figure S4).

A total of 53 known metabolites were selected in the elastic net regression for total red meat intake, 55 for unprocessed red meat, 36 for processed red meat, 7 for poultry, 18 for total fish, 27 for dark meat fish, and 11 for canned tuna fish (Table 2). Each metabolite profile score based on the selected known metabolites was significantly correlated with the corresponding self-reported dietary intake in both NHS/NHSII/HPFS and WHS (*r* = 0.33–0.46 for total red meat; *r* = 0.36–0.42 for unprocessed red meat; *r* = 0.19–0.33 for processed red meat; *r* = 0.12–0.21 for poultry; *r* = 0.31–0.40 for total fish; *r* = 0.32–0.42 for dark meat fish; and *r* = 0.14–0.22 for canned tuna fish) (Table 2). Adding unknown metabolites into the metabolite profile scores did not materially improve their correlations with the dietary intake (Supplementary Table S3). As a sensitivity analysis, we adopted the random forest algorithm to impute the missingness and re-derived the metabolomic profile score. The newly derived scores were highly correlated to the original scores, with a Pearson *r* = 0.98 for the total red meat score, 0.98 for the unprocessed red meat, 0.96 for the processed red meat score, 0.92 for the poultry score, 0.99 for the total fish score, 0.99 for the dark meat fish score, and 0.97 for the canned tuna fish score.

After adjusting for potential confounders, dietary intake of processed red meat was significantly associated with CRC risk. The pooled OR for 1 SD higher intake was 1.15 (95% CI: 1.03, 1.29) (Table 3). We did not observe consistent associations for dietary intake of three fish groups across NHS/HPFS and WHS. An inverse association for total fish and canned tuna fish was only found in NHS/HPFS and for dark meat fish in WHS. In contrast, metabolite profile scores for these three fish groups were inversely associated with CRC risk in both NHS/HPFS and WHS (Table 3). The pooled OR per 1 SD higher was 0.86 (95% CI: 0.77, 0.96) for total fish, 0.86 (95% CI: 0.77, 0.96) for dark meat fish, and 0.87 (95% CI: 0.78, 0.97) for canned tuna fish. No significant associations were observed for other meat intakes or metabolite profile scores. The results remained similar after adjusting for menopausal status or ethnicity in NHS/HPFS (data not shown). In the analysis by tumor site, we observed that the above associations were stronger for rectal cancer than those for colon cancer (Supplementary Table S4).

Among metabolites selected in metabolite profile scores of all three fish groups, five metabolites (C22:6 LPC, C22:6 LPE, C58:9 TAG, C60:12 TAG, and C38:7 plasmalogen) were positively correlated to fish intake but inversely associated with CRC risk; two metabolites (C20:4 LPE and C22:5 LPC) were inversely correlated to fish intake but positively associated with CRC risk (Supplementary Figure S5).

## 7. Discussion

Leveraging metabolomics data from four studies, we found systematic differences in plasma metabolite profiles according to various types of meat and fish consumption. We also developed metabolite profile scores that were correlated to the consumption of red meat, poultry, and fish. Higher metabolite profile scores for all three fish groups (total fish, dark meat fish, and canned tuna fish), rather than their self-reported dietary intake, were consistently associated with a lower CRC risk in both NHS/HPFS and WHS. These findings suggest the promising use of metabolomics in complementing traditional dietary assessments to evaluate the association between dietary exposure and health outcomes.

Consistent with previous studies, we found that consumption of all three red meat groups was positively correlated with several PE and PC plasmalogens, all of which have a low degree of unsaturation and contain acid moieties derived from animal tissues [14,29]. In contrast, total fish intake was positively correlated with highly unsaturated lipids, including TAGs, PCs, PEs, LPCs, LPEs, CEs, and plasmalogens. Fish intake, especially marine fish, is the principal dietary contributor of highly n-3 polyunsaturated fatty acids [30], and thus, are dietary sources for these lipids and their downstream metabolites.

Apart from lipid metabolites, we also observed several other metabolites correlated with meat and fish consumption. Creatine, an amino acid metabolite abundant in skeletal muscle [31], was positively correlated with the intake of red meat, poultry, and fish. Hydroxyproline, an amino acid that forms part of collagen [32], was positively correlated with red meat and poultry intake. Additionally, we found a positive correlation between poultry intake and ectoine (an α-amino acid), in line with one previous study [14]. Ectoine can be synthesized and released by several species in the genus *Halomonas* [33], the dominant microbial genus in the chicken embryo [34], which might lead to the presence of ectoine in chicken.

Most previous metabolomic studies mainly focused on known metabolites, which are only a small percent of the data obtained in a typical metabolomics measurement (~10%) [35]. In the present study, we analyzed the unknown metabolites and identified a set of unknown metabolites that were significantly correlated with meat or fish consumption. Several unknown metabolites even exhibited a higher correlation with meat and fish intake (partial Spearman *rho* > 0.3) compared to known metabolites. By further examining the correlation between these unknown metabolites and the known metabolites, strong correlations were found with C38:6 PC, C40:6 PC, C40:9 PC, and C22:6 LPC, indicating their potential biological links. However, whether these unknown metabolites are metabolites of the known metabolites or unrelated molecules affected by the same food is not clear. A critical next step is to structurally identify the promising and influential unknown metabolites. It should also be noted that despite the many statistically significant correlations between meat and fish groups and the unknown metabolites, these unknown metabolites added minimally to the correlations between meat and fish intakes and metabolite profile score based only on the known metabolites. The possibility remains that other metabolomics platforms may add further independent prediction of intake.

The human metabolome reflects a holistic biological status, which can be influenced by various exogenous and endogenous factors, such as dietary intakes, gut microbiota, health status, and genotype [36,37]. The metabolites selected in the metabolite profile score for meat or fish consumption could represent, in part, the components of food, such as the eicosapentaenoic acid/docosahexaenoic acid-containing lipids for the fish intake and hydroxyproline for the intake of red meat and poultry. Other metabolites, for example, C20:4 LPE and C22:5 LPC, could be related to the metabolic processes after fish intake, as fish is one of the main sources of arachidonic acid (C20:4) [38] and docosapentaenoic acid (C22:5) [39], but it was inversely correlated with these two lipids. Thus, metabolite profile score can be a comprehensive way to reflect both genuine compounds from foods as well as markers of complex metabolomic responses to dietary exposures.

Not surprisingly, we found a significant positive association between processed red meat intake and CRC risk. However, the metabolite profile score for processed red meat consumption was not significantly associated with CRC risk. One possible explanation could be that the increased CRC risk associated with high processed red meat intake was mainly due to the carcinogenic compounds [40], such as polycyclic aromatic hydrocarbons, heterocyclic amines, and N-nitroso compounds, which were not measured in our metabolomics platforms. On the contrary, we did not observe a consistent inverse association between dietary fish intake and CRC risk, but inverse associations were found for the metabolite profile scores of all three fish groups in both NHS/HPFS and WHS. Results from previous studies of fish intake and CRC risk were generally inconsistent, some indicating an inverse association [7,8], but others not [5,6]. Unlike self-reported dietary intake data widely used in previous studies, the metabolomics data objectively captures the metabolites related to dietary exposures in the human body, which might be more directly associated with health outcomes. The seven metabolites (C20:4 LPE, C22:5 LPC, C22:6 LPC, C22:6 LPE, C58:9 TAG, C60:12 TAG, and C38:7 plasmalogen) selected in metabolite profile scores of all three fish groups could represent the beneficial metabolomic response to fish intake, and this response led to a lower risk of CRC.

The main strengths of our study are the large sample size, comprehensive metabolite profiles including both known and unknown metabolites, prospectively collected dietary data before CRC diagnosis, and the use of the elastic net model, which performs well in high-dimensional data where there are high correlations between predictors [41]. Moreover, the robustness of our metabolite profile scores was validated in an independent study. Nevertheless, our results also have several limitations. First, we did not have data on the type of red meat. Red meat subtypes (beef, lamb, or pork) may differ in their associations with CRC risk [42]. Second, only one metabolomics measurement was performed for each participant. However, our previous pilot study indicated that most metabolites were highly stable over 1–2 years within individuals [26]. Third, the metabolites measured in our study focused on lipids, amino acids, and amino acid derivatives and did not include all established biomarkers of food intake. Finally, participants in our study were mainly white women; replication of our findings in men and other racial groups is needed.

In conclusion, we identified a panel of metabolites differentially correlated with the intake of red meat, poultry, and fish. We also developed metabolite profile scores associated with the consumption of meat and fish and observed consistent inverse associations between metabolite profile scores of fish intake and CRC risk. Our results suggest the potential utility of metabolomics in complementing traditional dietary assessments to investigate the diet-disease association and disentangle the metabolomic responses linking higher fish intake with reduced CRC risk.

## Figures and Tables

**Table 1:** Characteristics of the study participants in NHS, NHSII, and HPFS, and external replication participants in WHS.

	NHS, NHSII, and HPFS			WHS (Included in the Nested Case-Control Analysis of CRC)	
	Overall (*n* = 5269)	Included in the Nested Case-Control Analysis of CRC			
		Cases (*n* = 559)	Controls (*n* = 559)	Cases (*n* = 266)	Controls (*n* = 266)
Age at blood draw, years	53 (9)	61 (8)	61 (8)	59 (8)	59 (8)
Female, %	90	65	65	100	100
White race, %	98	97	99	98	98
BMI, kg/m^2^	25.4 (4.9)	25.9 (4.4)	25.5 (4.2)	26.7 (5.5)	26.2 (5.0)
Participants selected as cases in sub-studies, %	50	100	0	100	0
Fasting at blood collection, %	69	65	65	73	73
Multivitamin use, %	68	62	64	29	28
Regular aspirin use, %	42	47	49	10	13
Endoscopy, %	28	43	47	4 ^a^	3 ^a^
Family history of CRC, %	11	18	14	14	11
Smoking, %					
Never	54	45	46	45	54
Former	36	45	46	43	36
Current	10	10	8	12	10
Physical activity, MET-hours/week	18.4 (22.2)	19.9 (21.6)	21.2 (24.7)	14.4 (20.8)	16.5 (27.6)
Alternate Healthy Eating Index ^b^	24.7 (6.6)	25.3 (6.7)	25.5 (7.0)	/	/
Assigned to aspirin group (for WHS), %	/	/	/	43	49
Assigned to vitamin E group (for WHS), %	/	/	/	45	50
Dietary intake					
Total energy intake, kcal/day	1830 (506)	1873 (552)	1910 (543)	1692 (530)	1752 (538)
Alcohol intake, g/day	5.8 (10.4)	8.7 (14.5)	8.4 (12.9)	5.0 (9.9)	4.2 (7.6)
Total red meat intake, servings/week	6.6 (4.2)	7.1 (5.2)	6.8 (4.4)	6.1 (5.2)	6.5 (5.4)
Unprocessed red meat, servings/week	4.9 (3.2)	5.1 (3.8)	5.0 (3.3)	4.7 (3.7)	5.2 (3.9)
Processed red meat, servings/week	1.6 (1.8)	2.0 (2.3)	1.7 (1.9)	1.3 (1.8)	1.3 (1.8)
Poultry, servings/week	4.3 (2.6)	4.2 (2.9)	4.2 (2.3)	2.7 (1.8)	2.8 (1.8)
Total fish, servings/week	1.8 (1.6)	2.0 (1.5)	2.3 (2.1)	1.5 (1.4)	1.5 (1.4)
Dark meat fish, servings/week	0.3 (0.5)	0.3 (0.5)	0.4 (0.6)	0.2 (0.3)	0.2 (0.4)
Canned tuna fish, servings/week	0.9 (0.9)	0.9 (0.9)	1.1 (1.4)	0.7 (0.8)	0.7 (0.9)

Values are means (SDs) for continuous variables and percentages for categorical variables. BMI, body mass index; CRC, colorectal cancer; HPFS, Health Professional Follow-up Study; MET, metabolic equivalent task; NHS, Nurses’ Health Study; NHSII, Nurses’ Health Study II; WHS, Women’s Health Study. ^a^ Information was obtained at the first 12-month follow-up questionnaire after the trial. ^b^ Intakes of red meat, alcohol, and three types of fat (trans fat, long-chain n-3 fats, and polyunsaturated fats) were not included in the calculation, range is 0–60.

**Table 2:** Pearson correlation coefficients between meat and fish intake and the corresponding metabolite profile score.

	NHS, NHSII, HPFS (*n* = 5269)			WHS (*n* = 532)	
	Number of Known Metabolites in the Metabolite Profile Score	*r* (95% CI)		Number of Known Metabolites Available in the Score Calculation	*r* (95% CI)
		Training ^a^ (*n* = 3688)	Testing (*n* = 1581)		
Total red meat	53	0.44 (0.41, 0.46)	0.46 (0.42, 0.49)	50	0.33 (0.25, 0.40)
Unprocessed red meat	55	0.40 (0.38, 0.43)	0.42 (0.38, 0.46)	55	0.36 (0.28, 0.43)
Processed red meat	36	0.32 (0.29, 0.35)	0.33 (0.29, 0.38)	34	0.19 (0.12, 0.28)
Poultry	7	0.21 (0.18, 0.24)	0.18 (0.14, 0.23)	6	0.12 (0.04, 0.20)
Total fish	18	0.40 (0.38, 0.43)	0.39 (0.35, 0.43)	18	0.31 (0.23, 0.38)
Dark meat fish	27	0.42 (0.40, 0.45)	0.42 (0.38, 0.46)	25	0.32 (0.24, 0.39)
Canned tuna fish	11	0.22 (0.19, 0.25)	0.20 (0.15, 0.25)	11	0.14 (0.06, 0.22)

CI, confidence interval; HPFS, Health Professional Follow-up Study; NHS, Nurses’ Health Study; NHSII, Nurses’ Health Study II; WHS, Women’s Health Study. ^a^ Calculated using leave-one-out approach.

**Table 3:** Associations of meat and fish intake and the corresponding metabolite profile score with CRC risk.

	Dietary Intake			Metabolite Profile Score		
	NHS/HPFS (*n* = 1118)	WHS (*n* = 532)	Pooled	NHS/HPFS (*n* = 1118)	WHS (*n* = 532)	Pooled
Total red meat						
Basic model ^a^	1.09 (0.97, 1.24)	0.94 (0.80, 1.10)	1.03 (0.94, 1.14)	1.07 (0.95, 1.21)	1.16 (0.97, 1.39)	1.10 (0.99, 1.21)
Multivariable model ^b^	1.16 (0.99, 1.35)	0.95 (0.77, 1.16)	1.07 (0.95, 1.21)	1.02 (0.89, 1.16)	1.14 (0.95, 1.37)	1.06 (0.95, 1.18)
Unprocessed red meat						
Basic model ^a^	1.03 (0.91, 1.16)	0.91 (0.77, 1.08)	0.99 (0.89, 1.09)	1.05 (0.93, 1.18)	1.13 (0.95, 1.35)	1.08 (0.97, 1.19)
Multivariable model ^b^	1.05 (0.91, 1.21)	0.91 (0.74, 1.12)	1.00 (0.89, 1.13)	1.00 (0.87, 1.14)	1.12 (0.93, 1.34)	1.04 (0.93, 1.15)
Processed red meat						
Basic model ^a^	1.18 (1.04, 1.34)	1.01 (0.85, 1.18)	1.11 (1.00, 1.23)	1.12 (0.99, 1.27)	1.18 (0.98, 1.41)	1.14 (1.03, 1.26)
Multivariable model ^b^	1.23 (1.06, 1.42)	1.03 (0.85, 1.25)	1.15 (1.03, 1.29)	1.07 (0.93, 1.22)	1.16 (0.95, 1.40)	1.10 (0.98, 1.22)
Poultry						
Basic model ^a^	1.02 (0.91, 1.15)	0.91 (0.77, 1.08)	0.99 (0.90, 1.09)	0.99 (0.88, 1.12)	0.87 (0.73, 1.04)	0.95 (0.86, 1.05)
Multivariable model ^b^	1.07 (0.94, 1.22)	0.94 (0.78, 1.12)	1.02 (0.92, 1.14)	0.99 (0.87, 1.12)	0.86 (0.72, 1.04)	0.94 (0.85, 1.05)
Total fish						
Basic model ^a^	0.82 (0.71, 0.95)	1.00 (0.84, 1.19)	0.89 (0.79, 0.99)	0.87 (0.76, 0.98)	0.85 (0.71, 1.02)	0.86 (0.78, 0.95)
Multivariable model ^b^	0.84 (0.72, 0.98)	1.06 (0.88, 1.29)	0.92 (0.82, 1.04)	0.87 (0.77, 0.99)	0.84 (0.70, 1.02)	0.86 (0.77, 0.96)
Dark meat fish						
Basic model ^a^	0.96 (0.85, 1.09)	0.86 (0.72, 1.03)	0.93 (0.84, 1.03)	0.86 (0.76, 0.98)	0.85 (0.71, 1.02)	0.86 (0.78, 0.95)
Multivariable model ^b^	0.98 (0.86, 1.11)	0.86 (0.71, 1.04)	0.94 (0.85, 1.05)	0.87 (0.76, 0.99)	0.84 (0.70, 1.03)	0.86 (0.77, 0.96)
Canned tuna fish						
Basic model ^a^	0.80 (0.69, 0.93)	0.98 (0.83, 1.17)	0.87 (0.78, 0.98)	0.87 (0.77, 0.99)	0.87 (0.72, 1.04)	0.87 (0.79, 0.96)
Multivariable model ^b^	0.82 (0.70, 0.95)	1.00 (0.83, 1.20)	0.89 (0.79, 1.00)	0.88 (0.77, 1.00)	0.87 (0.72, 1.05)	0.87 (0.78, 0.97)

Odds ratio (OR) and 95% confidence interval (CI) of CRC risk per standard deviation increment in dietary intakes or the metabolite profile scores were presented. ^a^ The basic models were conducted using conditional logistic regression without adjusting for any covariates. ^b^ The multivariable models were further adjusted for BMI, family history of CRC, endoscopy, multivitamin use, aspirin use, smoking, physical activity, total energy intake, alcohol intake, and modified AHEI (in NHS/HPFS). AHEI, Alternate Healthy Eating Index; BMI, body mass index; CRC, colorectal cancer; HPFS, Health Professional Follow-up Study; NHS, Nurses’ Health Study; WHS, Women’s Health Study.

## Data Availability

The data underlying this article will be shared on reasonable request to the corresponding author.

## Supplementary Materials

The following supporting information can be downloaded at: https://www.mdpi.com/article/10.3390/nu14050978/s1, Figure S1: Categories (A) and marginal Pearson correlation (B) of the 287 known metabolites included in the analysis; Figure S2: Heatmap of known metabolites significantly associated with meat and fish intake, restricted to fasting participants; Figure S3: Heatmap of known metabolites significantly associated with meat and fish intake, restricted to control participants; Figure S4: Pearson correlations between unknown metabolites strongly correlated to meat and fish intake and known metabolites; Figure S5: Known metabolites selected in the metabolite profile scores and their associations with CRC; Table S1: Serving sizes for each food group; Table S2: Number of unknown metabolites significantly associated with meat and fish intake; Table S3: Pearson correlation coefficients between dietary consumption and metabolite profile scores of meat and fish consumption after adding unknown metabolites into the score; Table S4: Associations of meat and fish intake and the corresponding metabolite profile score with risk of colon cancer and rectal cancer in the Nurses’ Health Study/Health Professional Follow-up Study.
