# Supplementary material for: Plasma Metabolite Profiles of Red Meat, Poultry, and Fish Consumption, and Their Associations with Colorectal Cancer Risk
Source: Nutrients. 2022 Feb 25;14(5):978. doi: 10.3390/nu14050978 (PMC8912563; doi:10.3390/nu14050978)
Supplement: Supplementary file 1 [file nutrients-14-00978-s001.zip › nutrients-1582445-supplementary.pdf]

Supplementary Table S1. Serving sizes for each food group.

	One serving size
Unprocessed red meat	
Hamburgers	85 g
Beef, pork, or lamb as a sandwich or mixed dish	85 g
Beef, pork, or lamb as a main dish	85 g
Processed red meat	
Bacon	28 g for two slices
Hot dogs	45 g
Sausage, salami, bologna, or other processed meat	45 g for one piece
Poultry	
Chicken or turkey with or without skin	85 g
Chicken or turkey sandwiches	85 g
Chicken or turkey hot dogs	45 g
Fish	
Dark meat fish (e.g., salmon)	85-140 g
Canned tuna fish	85-112 g
Breaded fish cakes, pieces, or fish sticks	One piece/stick
Other fish	85-140 g

Supplementary Table S2. Number of unknown metabolites significantly associated with meat and fish intake.

**Meat and fish groups**	***rho*** **[-0.3, -0.2)**	***rho*** **[-0.2, -0.1)**	***rho*** **[-0.1, 0)**	***rho*** **(0, 0.1]**	***rho*** **(0.1, 0.2]**	***rho*** **(0.2, 0.3]**	***rho*** **(0.3, 0.4]**
Total red meat	1	80	175	175	250	20	1
Unprocessed red meat	0	7	57	98	54	6	0
Processed red meat	0	17	104	124	216	1	0
Poultry	0	0	7	46	12	0	0
Total fish	2	30	80	50	60	52	10
Dark meat fish	1	37	191	54	67	59	5
Canned tuna fish	0	4	17	30	68	0	0

Correlation coefficients were calculated by partial Spearman correlation analyses adjusting for age at blood draw, fasting status, endpoints and case/control status in the original sub-study, BMI, smoking status, physical activity, alcohol intake, total energy intake, and modified AHEI. These meat and fish groups were also mutually adjusted. Significance was defined as $P < 0.05$ after Bonferroni correction. AHEI, Alternate Healthy Eating Index; BMI, body mass index.

Supplementary Table S3. Pearson correlation coefficients between dietary consumption and metabolite profile scores of meat and fish consumption after adding unknown metabolites into the score.

	**No. of known metabolites selected**	r^a	**No. of unknown metabolites added**	r^b	P^c
Total red meat	53	0.46	55	0.51	<0.001
Unprocessed red meat	55	0.42	34	0.48	<0.001
Processed red meat	36	0.33	6	0.36	<0.001
Poultry	7	0.18	11	0.19	0.61
Total fish	18	0.39	4	0.41	0.01
Dark meat fish	27	0.42	1	0.43	0.67
Canned tuna fish	11	0.20	1	0.21	0.27

As shown in Figure 1, analysis was conducted only in the testing set (n = 1581). [a]Based on metabolite profile scores just using known metabolites. [b]Based on metabolite profile scores including both known and unknown metabolites. [c]P for comparison of the two correlated correlation coefficients using test developed by Meng, Rosenthal, and Rubin.

Supplementary Table S4. Associations of meat and fish intake and the corresponding metabolite profile score with risk of colon cancer and rectal cancer in the Nurses' Health Study/Health Professional Follow-up Study.

	**Colon cancer (*n* = 808)**		**Rectal cancer (*n* = 244)**	
	**Dietary intake**	**Metabolite profile score**	**Dietary intake**	**Metabolite profile score**
Total red meat				
Basic model[a]	1.16 (1.00, 1.34)	1.07 (0.92, 1.23)	1.12 (0.85, 1.46)	1.20 (0.92, 1.56)
Multivariable model[b]	1.21 (1.01, 1.46)	0.99 (0.84, 1.16)	1.21 (0.84, 1.74)	1.20 (0.86, 1.66)
Unprocessed red meat				
Basic model[a]	1.11 (0.96, 1.28)	1.05 (0.91, 1.21)	0.98 (0.75, 1.27)	1.16 (0.89, 1.51)
Multivariable model[b]	1.13 (0.94, 1.34)	0.98 (0.84, 1.14)	1.00 (0.72, 1.39)	1.15 (0.84, 1.58)
Processed red meat				
Basic model[a]	1.17 (1.01, 1.35)	1.12 (0.97, 1.29)	1.39 (1.01, 1.91)	1.24 (0.94, 1.62)
Multivariable model[b]	1.20 (1.01, 1.41)	1.04 (0.89, 1.22)	1.57 (1.03, 2.39)	1.24 (0.89, 1.73)
Poultry				
Basic model[a]	1.00 (0.87, 1.15)	0.98 (0.84, 1.13)	0.95 (0.74, 1.21)	1.01 (0.79, 1.31)
Multivariable model[b]	1.03 (0.88, 1.20)	0.97 (0.83, 1.13)	0.88 (0.66, 1.17)	0.90 (0.66, 1.22)
Total fish				
Basic model[a]	0.85 (0.71, 1.01)	0.86 (0.74, 1.00)	0.71 (0.54, 0.94)	0.83 (0.64, 1.09)
Multivariable model[b]	0.87 (0.73, 1.05)	0.87 (0.75, 1.02)	0.63 (0.45, 0.89)	0.74 (0.54, 1.02)
Dark meat fish				
Basic model[a]	0.96 (0.83, 1.12)	0.86 (0.74, 0.99)	1.02 (0.81, 1.29)	0.82 (0.63, 1.07)
Multivariable model[b]	0.99 (0.85, 1.15)	0.87 (0.74, 1.02)	1.01 (0.78, 1.32)	0.74 (0.55, 1.02)
Canned tuna fish				
Basic model[a]	0.88 (0.74, 1.04)	0.88 (0.76, 1.02)	0.56 (0.38, 0.82)	0.80 (0.61, 1.06)
Multivariable model[b]	0.89 (0.75, 1.06)	0.89 (0.76, 1.04)	0.51 (0.33, 0.80)	0.70 (0.51, 0.98)

Odds ratio and 95% confidence interval of colon cancer and rectal cancer risk per standard deviation increment in dietary intakes or the metabolite profile scores were presented. [a]The basic models were conducted using conditional logistic regression without adjusting for any covariates. [b]The multivariable models were further adjusted for body mass index, family history of colorectal cancer, endoscopy, multivitamin use, aspirin use, smoking, physical activity, total energy intake, alcohol intake, and modified Alternate Healthy Eating Index.

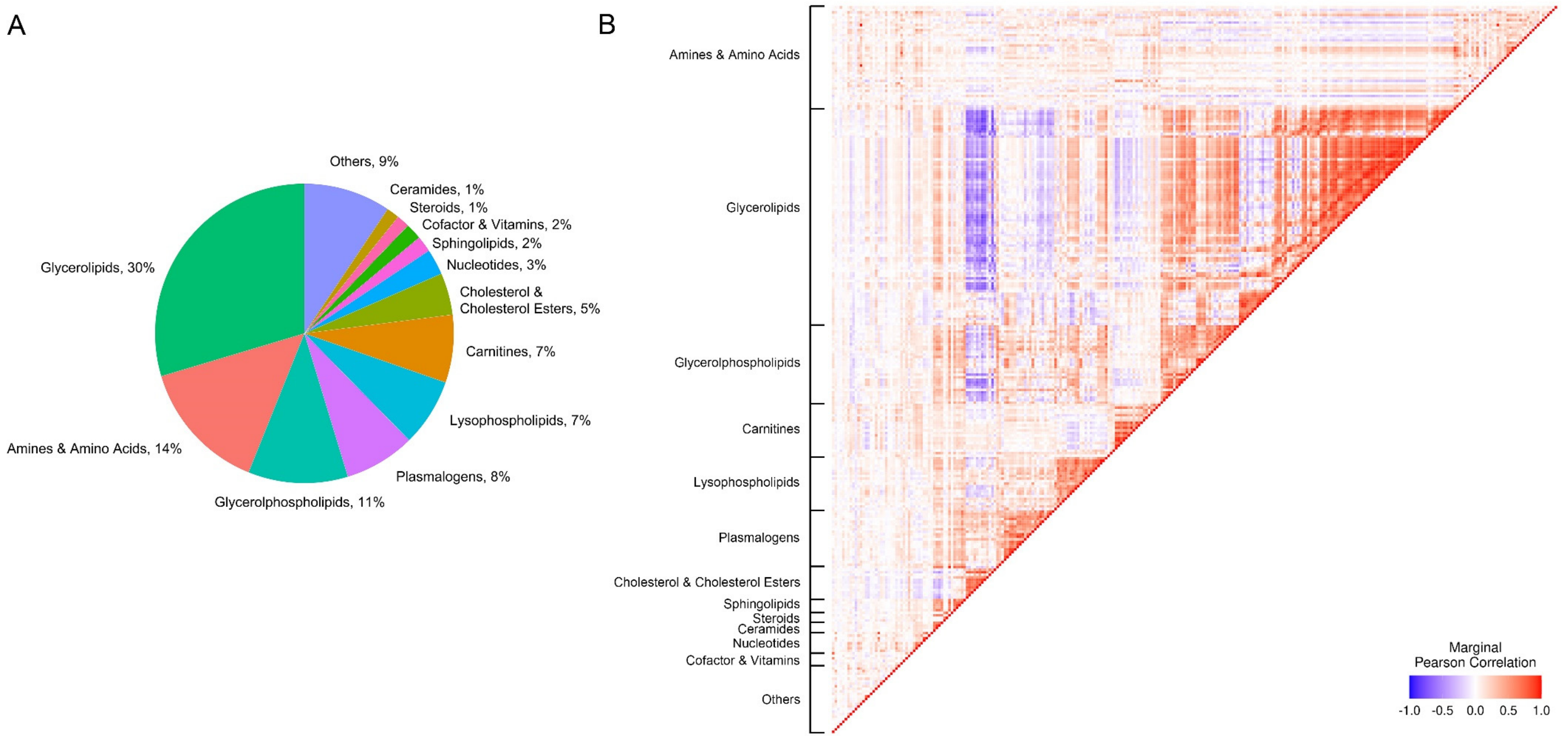

Supplementary Figure S1. Categories (A) and marginal Pearson correlation (B) of the 287 known metabolites included in the analysis**.**

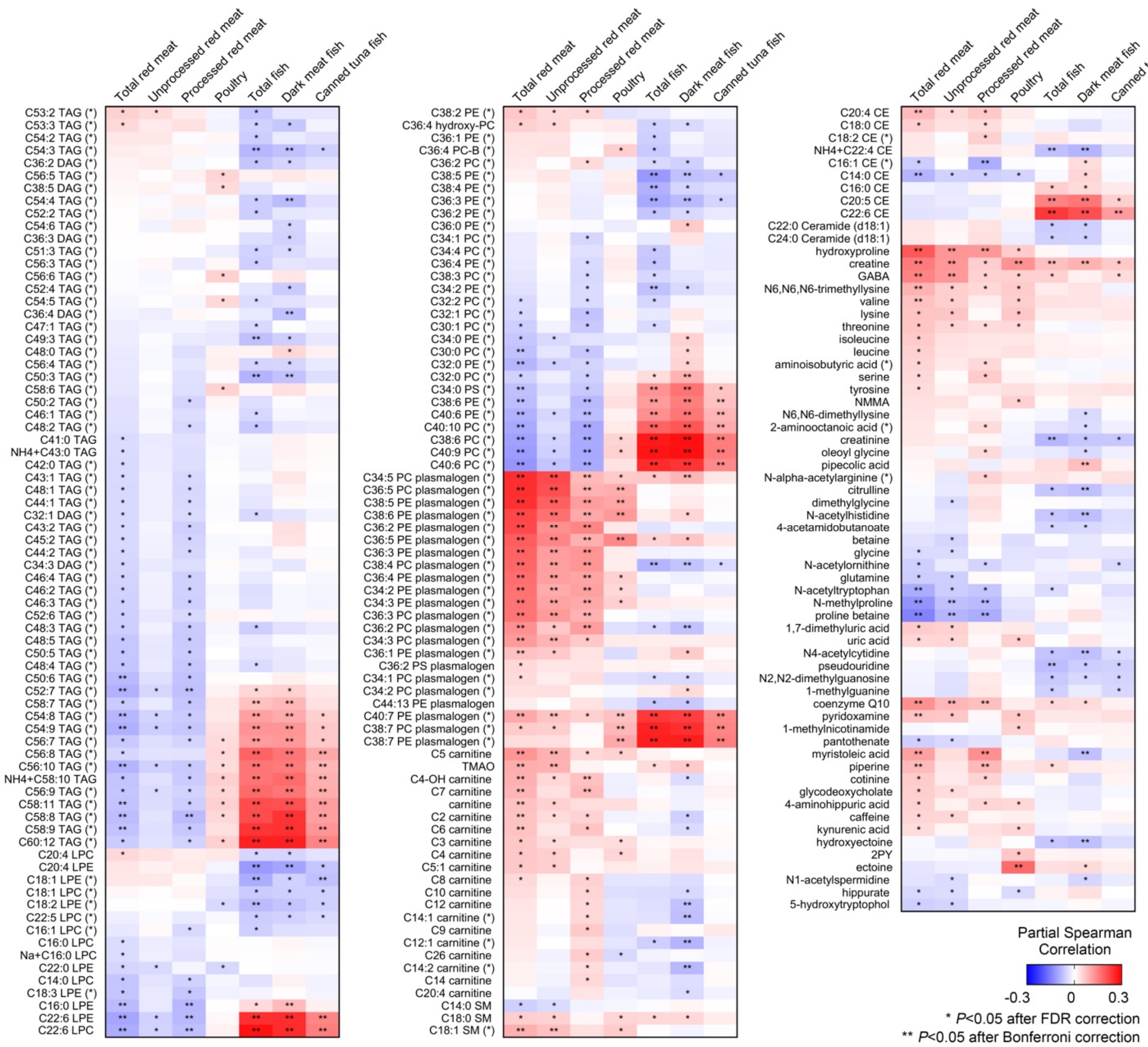

Supplementary Figure S2. Heatmap of known metabolites significantly associated with meat and fish intake, restricted to fasting participants**.** Analysis was conducted among fasting participants (n = 3640). Only metabolites significantly (FDR correction) correlated with at least one meat or fish group are shown. The intensity of the colors represents the degree of association between plasma metabolites and consumption of total red meat, unprocessed red meat, processed red meat, poultry, total fish, dark meat fish, and canned tuna fish, as measured by partial Spearman correlation analyses adjusting for age at blood draw, endpoints and case/control status in the original sub-study, BMI, smoking status, physical activity, alcohol intake, total energy intake, and modified AHEI. These meat and fish groups were also mutually adjusted. Metabolite with "(*)" indicate a representative name. *, P < 0.05 after FDR correction and **, P < 0.05 after Bonferroni correction. AHEI, Alternate Healthy Eating Index; BMI, body mass index.

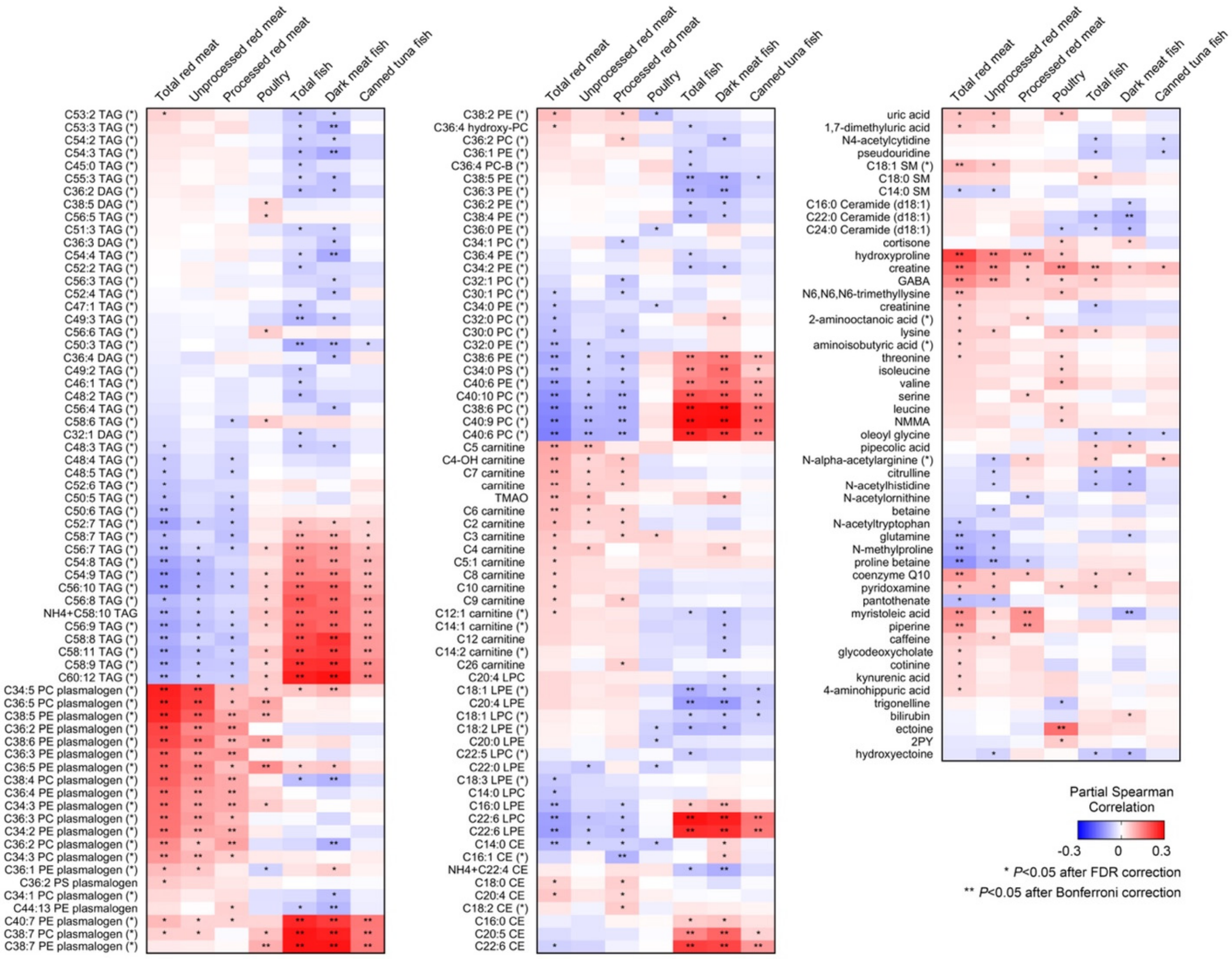

Supplementary Figure S3. Heatmap of known metabolites significantly associated with meat and fish intake, restricted to control participants. Analysis was conducted among participants selected as controls in the original sub-studies (*n* = 2624). Only metabolites significantly (FDR correction) correlated with at least one meat or fish group are shown. The intensity of the colors represents the degree of association between plasma metabolites and consumption of total red meat, unprocessed red meat, processed red meat, poultry, total fish, dark meat fish, and canned tuna fish, as measured by partial Spearman correlation analyses adjusting for age at blood draw, fasting status, endpoints in the original sub-study, BMI, smoking status, physical activity, alcohol intake, total energy intake, and modified AHEI. These meat and fish groups were also mutually adjusted. Metabolite with "(*)" indicate a representative name. *, $P < 0.05$ after FDR correction and **, $P < 0.05$ after Bonferroni correction. AHEI, Alternate Healthy Eating Index; BMI, body mass index.

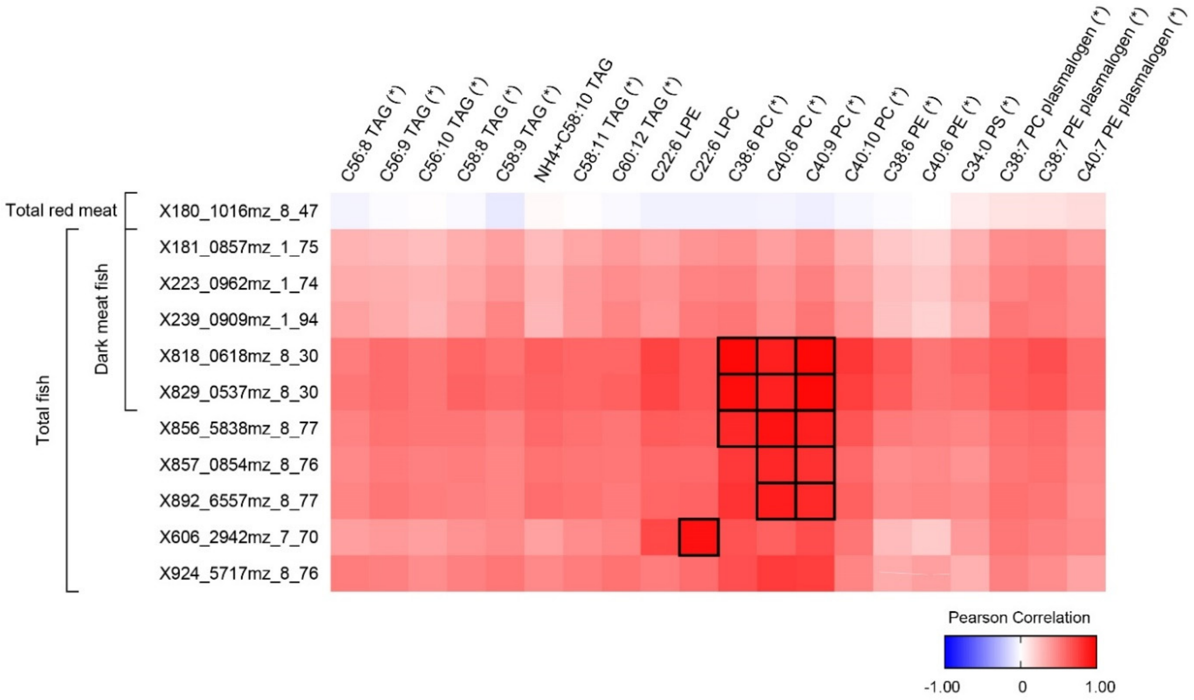

Supplementary Figure S4. Pearson correlations between unknown metabolites strongly correlated to meat and fish intake and known metabolites. The presented 11 unknown metabolites were those having a relative strong correlation (absolute partial Spearman *rho* > 0.3) with intake of meat and fish, specifically, total red meat, total fish, and dark meat fish. The presented known metabolites (n = 20) were those significantly associated with intake of total red meat, total fish, and dark meat fish after Bonferroni correction and have a relative strong correlation (absolute Pearson r > 0.5) with at least one unknown metabolite. The cell with a border indicates a Pearson correlation coefficient > 0.8. Known metabolite with "(*)" indicate a representative name.

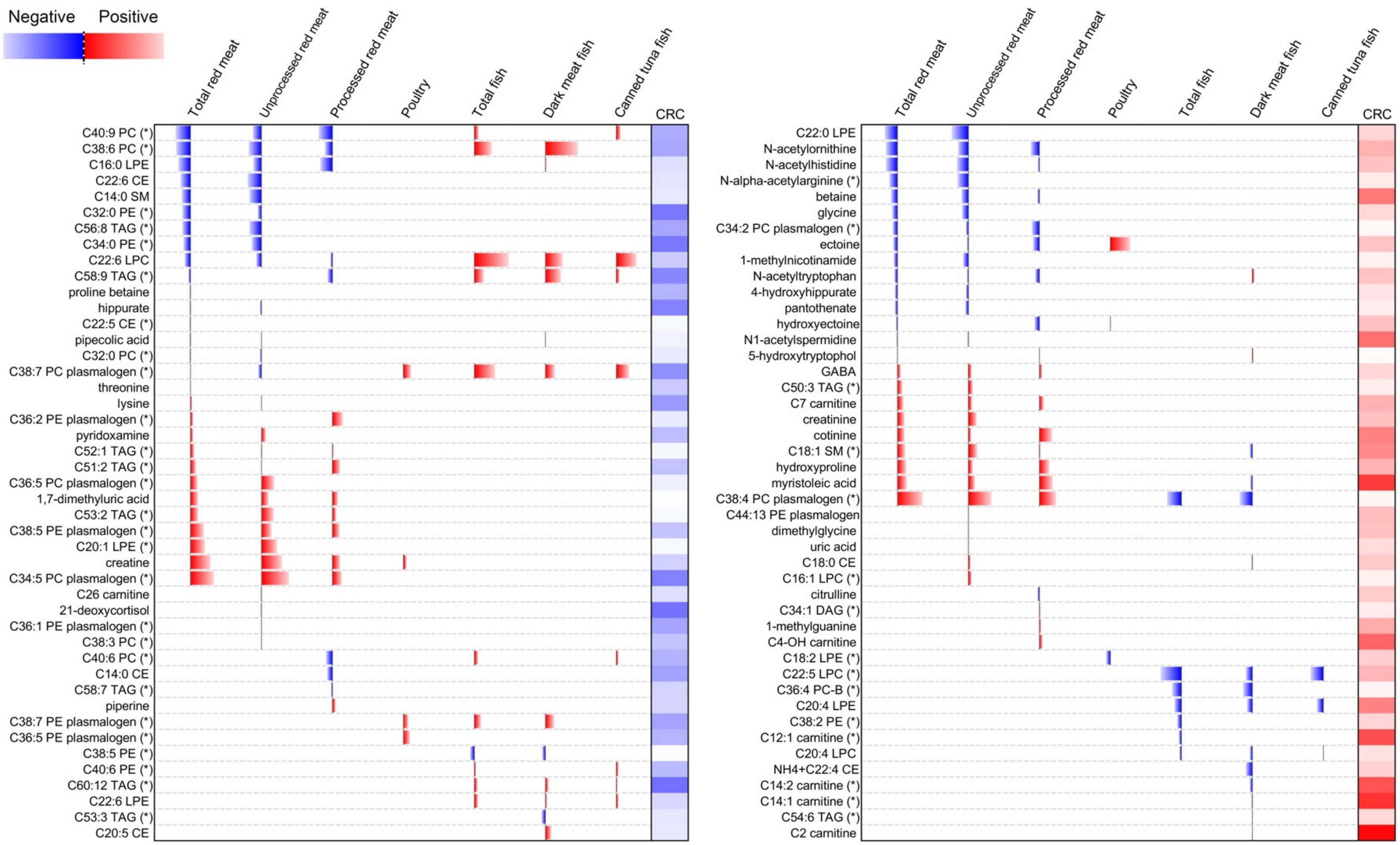

Supplementary Figure S5. Known metabolites selected in the metabolite profile scores and their associations with CRC. The left panel shows the metabolites selected in the metabolite profile scores and negatively associated with CRC risk; the panel right shows the metabolites selected in the metabolite profile scores and positively associated with CRC risk. Within each panel, presented from left to right are the metabolites' coefficients (weights) in the corresponding score, and the ln (odds ratio) of CRC risk per 1-SD increment in metabolites. Color denotes the direction of association (red-positive and blue-inverse) and magnitude (the darker the color, the stronger the magnitude). Metabolite with "(*)" indicate a representative name. CRC, colorectal cancer.
